# An efficient rRNA removal method for RNA sequencing in GC-rich bacteria

**DOI:** 10.1186/2042-5783-3-1

**Published:** 2013-01-07

**Authors:** Clelia Peano, Alessandro Pietrelli, Clarissa Consolandi, Elio Rossi, Luca Petiti, Letizia Tagliabue, Gianluca De Bellis, Paolo Landini

**Affiliations:** 1 Institute of Biomedical Technologies, National Research Council, Segrate, Milan, Italy; 2 Department of Biosciences, University of Milan, Milan, Italy; 3 Department of Medical Biotechnologies and Translational Medicine, University of Milan, Milan, Italy

**Keywords:** rRNA removal, RNA-sequencing, GC-rich bacteria, Transcriptome analysis

## Abstract

**Background:**

Next generation sequencing (NGS) technologies have revolutionized gene expression studies and functional genomics analysis. However, further improvement of RNA sequencing protocols is still desirable, in order to reduce NGS costs and to increase its accuracy. In bacteria, a major problem in RNA sequencing is the abundance of ribosomal RNA (rRNA), which accounts for 95-98% of total RNA and can therefore hinder sufficient coverage of mRNA, the main focus of transcriptomic studies. Thus, efficient removal of rRNA is necessary to achieve optimal coverage, good detection sensitivity and reliable results. An additional challenge is presented by microorganisms with GC-rich genomes, in which rRNA removal is less efficient.

**Results:**

In this work, we tested two commercial kits for rRNA removal, either alone or in combination, on *Burkholderia thailandensis*. This bacterium, chosen as representative of the important *Burkholderia* genus, which includes both pathogenic and environmental bacteria, has a rather large (6.72 Mb) and GC-rich (67.7%) genome. Each enriched mRNA sample was sequenced through paired-end Illumina GAIIx run in duplicate, yielding between 10 and 40 million reads. We show that combined treatment with both kits allows an mRNA enrichment of more than 238-fold, enabling the sequencing of almost all (more than 90%) *B. thailandensis* transcripts from less than 10 million reads, without introducing any bias in mRNA relative abundance, thus preserving differential expression profile.

**Conclusions:**

The mRNA enrichment protocol presented in this work leads to an increase in detection sensitivity up to 770% compared to total RNA; such increased sensitivity allows for a corresponding reduction in the number of sequencing reads necessary for the complete analysis of whole transcriptome expression profiling. Thus we can conclude that the MICROBExpress/Ovation combined rRNA removal method could be suitable for RNA sequencing of whole transcriptomes of microorganisms with high GC content and complex genomes enabling at the same time an important scaling down of sequencing costs.

## Background

The advent of functional genomics and the availability of next generation sequencing (NGS) technologies have dramatically changed the approach to studying gene expression. Massive RNA sequencing provides a detailed snapshot of the total RNA (the “transcriptome”) present at a given time in a cell. The transcriptome comprises coding (mRNA) and non-coding RNA (rRNA, tRNA, regulatory RNA and other RNA species). Quantitative differences in the gene expression patterns, either between cells grown in different conditions or between a mutant and its parental strain, can be identified in their entirety through this approach. However, in order to obtain reliable information, it is crucial that RNA sequencing achieves a sufficient “coverage”, enough to detect even rare RNA species. In bacterial cells, a disadvantage of the transcriptomic approach is the high amount of ribosomal RNA (rRNAs), which accounts for more than 95% of total cellular RNA, greatly reducing useful transcript coverage. Thus, efficient removal of rRNA is critical for successful transcriptome profiling. Unlike for eukaryotic mRNA, polyadenylation of bacterial mRNAs is limited and is mostly involved in targeting mRNA for degradation by PNPase [1]; hence, bacterial mRNA cannot be readily isolated from other RNA sources by hybridization to immobilized poly-T or enriched through reverse transcription with poly-T primers. Therefore, a major challenge in RNA-seq applications in bacterial cells is the enrichment for all transcript species other than rRNA and tRNA.

Papers describing the use of high-throughput sequencing for transcriptomics in bacteria have used mRNA enrichment methods usually based on depletion of rRNA and other RNAs [2-4], utilizing two alternative approaches: (i) hybridization capture of rRNAs by antisense oligonucleotides followed by pull down through binding to magnetic beads, (ii) degradation of processed RNA such as mature rRNA and tRNA by a 5′–3′ exonuclease that specifically digests RNA species with a 5′-monophosphate end.

The rRNA capture approach using the MICROBExpress Kit (Ambion) has widely been applied in RNA-seq studies [3-8], including metatranscriptomics [9,10]. Usage of the RiboMinus Bacteria Transcriptome Isolation Kit (Invitrogen), based on the same method, has only been reported in one study so far [11]. The rRNA capture approach is the only method suitable for precise quantitative analysis. However, as it is based on 16S and 23S rRNA specific capture probes, depletion efficiency of these kits varies between bacterial species. An alternative approach implemented a subtractive hybridization protocol using probes targeting bacterial, archaeal and eukaryotic fractions of environmental rRNA pools [12]. Finally, a recent work [13] reports a very extensive comparative analysis of five different rRNA removal methods, emphasizing the efficiency of the Ribo-Zero rRNA removal kit from Epicentre. The Ribo-Zero kit, also based on rRNA capture, proved to be very efficient both on pure cultures and on faecal samples, while preserving mRNAs relative abundance.

Alternatively to the capture-based methods, rRNA removal can also be achieved through its degradation by specific enzymes. An example is the mRNA-ONLY Prokaryotic mRNA Isolation Kit (Epicentre Biotechnologies), based on selective degradation of processed RNAs by the enzyme terminator 5′-phosphate-dependent exonuclease (TEX). This enzyme exclusively degrades RNA molecules carrying a 5′-monophosphate, *i.e.*, processed RNA such as rRNA and tRNA, while mRNAs, carrying a 5′-triphosphate group, are not affected [14]. This method can be useful for the analysis of very complex samples by RNA-seq (*e.g.*, environmental metatranscriptomics) [10], but it only provides semi-quantitative evaluation of gene expression levels. In some instances, selective rRNA methods have been used in combination with subtractive hybridization to optimize rRNA removal [15,16]. An additional advantage of rRNA degradation with TEX consists in the enrichment of primary transcripts with 5′-triphosphate ends, thus allowing identification of transcription start sites [14,17,18]. This methodology, termed differential RNA-seq (dRNA-seq), is extremely informative for promoter mapping and identification of small RNAs. Finally, another enzymatic method for mRNA enrichment that makes use of duplex-specific nuclease (DSN) to remove rRNA has been applied with good results, both in terms of mRNA coverage and robustness of mRNAs relative abundances, in transcriptome profiling of *Escherichia coli* grown in four different conditions [19].

In this work we tested the Ovation Prokaryotic RNA-Seq System kit for bacteria ribosomal RNA removal developed by NuGEN (NuGEN Technologies, San Carlos, CA, USA). Unlike the approaches described so far, which are based on rRNA removal or degradation, the Ovation kit relies on the synthesis of first and second strand cDNA using a random primer mix selectively designed to enrich the mRNA portion of bacterial total RNA. The selective random primers are designed against a sequence database composed of 50 bacterial and archaeal strains representing all of the major phylogenetic subgroups. The predicted binding site density of these primers on target (mRNA) and non-target (rRNA) transcripts is nearly identical across these species. The resulting cDNA is compatible with NuGEN’s Encore™ NGS Library Systems as well as other library workflows using double-stranded cDNA as input for the creation of sequencing libraries.

This new method was tested either in the absence of further treatments or in combination with an rRNA capture-based approach, *i.e.*, the MICROBExpress Kit from Ambion. A comparison of the results obtained from rRNA removal procedures based on different chemistries (capture with probes versus retro-transcription with selective random primers) and from libraries prepared with different protocols (Illumina TruSeq RNA libraries versus NuGEN’s Encore™ NGS libraries) was performed in order to evaluate the efficiency of the two methods, either alone or in combination, and to test the robustness of the protocols. The rRNA removal efficiency of the two kits, either separately or in combination was evaluated on RNA extracted from *Burkholderia thailandensis* in two different growth conditions. This bacterium was chosen because of its importance as a model organism for pathogenic species of *Burkholderia* such as *B. pseudomallei,* the etiological agent of melioidosis [20], and since its genome is characterized by a high GC content.

One of the main goals of this work was the reduction of sequencing costs: to this aim, we evaluated whether the two rRNA removal treatments, tested either separately or in combination, could result in a scaling down of the sequencing size (total reads produced) while preserving the whole transcriptome coverage. Our results show that the combination of the two kits leads to optimal results in terms of rRNA removal, without introducing a significant bias on relative mRNA abundances, and allow the sequencing of a GC rich bacteria transcriptome with less than 10 millions reads.

## Results

### Experimental design

In this comparative study, the *Burkholderia thailandensis* strain E264 (BtE264), a bacterium with a highly complex genome, both in terms of length/organization and of GC content, was used to test the mRNA enrichment efficiency by two different ribosomal RNA removal methods. RNA was extracted from *B. thailandensis* E264 cultures in stationary phase, grown ca. 20 hours in LB medium either in full aeration or in oxygen-limiting conditions.

Total RNA from *B. thailandensis* E264 was subjected to ribosomal RNA removal treatment using either the MICROBExpress Bacterial mRNA Enrichment Kit (Mex) or the Ovation Prokaryotic RNA-seq System (Ov) separately, or a combination of the two kits (Mex-Ov). Total RNA, not subjected to any rRNA removal procedure, was used as a control in transcriptome analysis. The RNA quality, measured using RNA electropherograms by Agilent 2100 Bioanalyzer, showed that the total RNA extracted was of good quality, with an RNA Integrity Number (RIN) higher than 9.0. The disappearance of the rRNA peaks, after the rRNA removal process, was also clearly detectable from the electropherograms.

For total RNA and for samples processed with the MICROBExpress kit only, the sequencing libraries were obtained using the Illumina TruSeq RNA library preparation kit, while the sequencing libraries for the RNA samples treated either with the Ovation kit or with the Mex/Ov combination were constructed using NuGEN’s Encore™ NGS Library System. Due to utilization of different methods, the sequencing libraries differ in the length of generated fragments: Illumina TruSeq RNA libraries are characterized by an average fragment length of 260bp, NuGEN’s Encore™ NGS libraries show an average fragment length of 160bp. Due to their shorter length we performed 48-cycle sequencing runs on NuGEN’s Encore™ NGS libraries, opposed to 86-cycle runs on Illumina TruSeq RNA libraries.

The profiles of the sequencing libraries prepared with the NuGEN’s Encore™ NGS system are shown in Additional file 1: Figure S1. It is noteworthy that the libraries obtained with the NuGEN’s Encore™ NGS kit display a distinct peak of about 130bp in the case of libraries characterized by a poor yield and likely due to the formation of adaptor ligation dimers. To avoid that such artefacts could negatively affect the quality of the reads obtained, all the reads found to be contaminated by Illumina adapters were excluded from further analysis; this filtering resulted in a reduction of about 10% of mapping reads in samples treated either with Ovation or with the combined treatment.

In order to test the reproducibility of the rRNA removal methods, each sample was prepared as a replicate and sequenced (Table 1); the sequencing libraries derived from the samples prepared by combining the two kits were sequenced twice to test the reproducibility of Illumina sequencing on such samples. Pearson’s correlation coefficient for sequencing replicate was higher than 0.99, indicating very high reproducibility of these sequencing data (Additional file 2: Figure S2).

**Table 1:** Sequencing reads and alignment statistics

**Sample name**	**Total reads**		**Mapped reads**		**Unmapped**		**% rRNA reads**		**% mRNA reads**		**% intergenic reads**	
	**Replicate 1**	**Replicate 2**	**Replicate 1**	**Replicate 2**	**Replicate 1**	**Replicate 2**	**Replicate 1**	**Replicate 2**	**Replicate 1**	**Replicate 2**	**Replicate 1**	**Replicate 2**
Bt-totRNA	4334132	3756060	41390060	36228341	1944072	1367719	99.63%	99.95%	0.29%	0.03%	0.08%	0.02%
B-t-Mex	26092578	29736910	24866950	28271413	1225628	1465497	96.57%	99.06%	2.82%	0.49%	0.61%	0.45%
Bt-Ov	13507220	15321918	10279802	11899910	3227418	3422008	52.48%	70.13%	41.15%	24.51%	6.37%	5.37%
Bt-Mex-Ov	10377152	7905258	7970400	6382124	2406752	1523134	62.57%	45.73%	30.04%	46.11%	7.39%	8.16%

More than 40 million reads were generated for total RNA samples. For samples processed for rRNA removal we progressively reduced the number of reads generated to evaluate if the Mex and Ov treatments, separately and in combination, allowed a scaling down of the sequencing size while preserving the whole transcriptome coverage. This scaling down was obtained by increasing the multiplexing of different samples in the same lane. An average of 28 million reads for the samples treated with MICROBExpress kit, of 14 million reads for the samples processed with Ovation and an average of 9 million reads for the samples subjected to double treatment were generated (Table 1).

### rRNA removal efficiency

**Figure 1:**
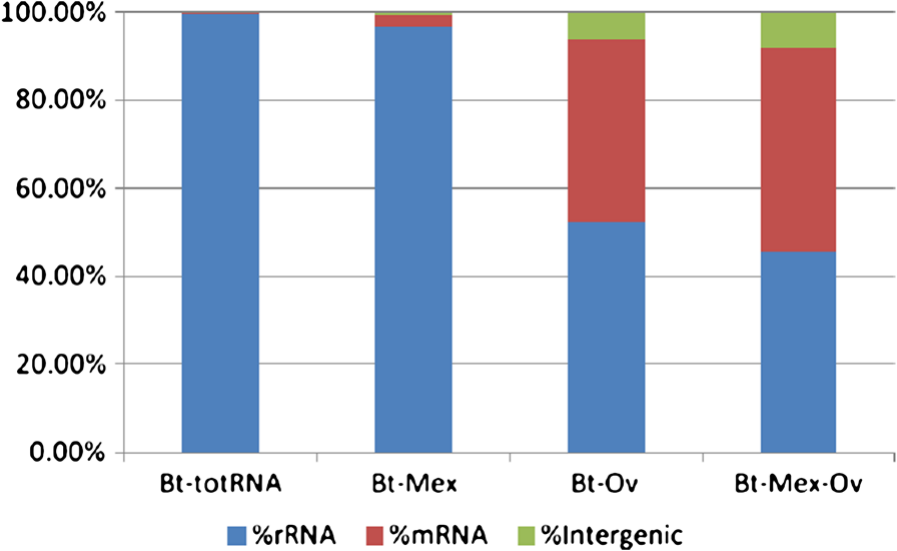
**rRNA removal efficiency.** Histogram showing the rRNA removal efficiency of the two methods applied separately and in combination. The percentage of reads mapping in rRNAs, in CDSs and in intergenic sequences are shown respectively in red, blue and green.

As expected, the Illumina deep sequencing of the control RNAs revealed that rRNA was the major component, with more than 99% of the reads mapping on rRNA gene sequences. The percentage of rRNA was only slightly affected by treatment with the MICROBExpress Kit (96% and 99% in the two runs), while it was reduced to an average of 61% using the Ovation kit, and to an average of 54% using the combined treatment (Figure 1). Conversely, the relative amount of mRNA increased significantly: mRNA enrichment ranged from about 10-fold using the MICROBExpress kit, to 205-238-fold with the Ovation treatment and with the combined treatment respectively (Table 1, Figure 1). It is noteworthy that Ov and Mex-Ov treatments increase the portion of reads mapping in intergenic regions respectively to 5.8% and 7.7% (Table 1, Figure 1). These reads could be crucial for the identification of non-coding and small RNAs that, although expressed at low levels, might be important in gene expression regulation. The analysis of these transcripts would be almost impossible in total RNA or in samples treated only with Mex because the percentage of reads mapping in intergenic regions is too low (0.05% and 0.53%, respectively).

### Improvement in mRNA detection sensitivity

Results shown in the previous sections clearly indicate that any rRNA removal treatment results in an increase of the percentage of mRNA; to determine whether such increase also improves mRNA detection sensitivity, the percentage of mRNA transcripts that are detected above a statistically significant threshold was evaluated. In RNA-Seq experiments, the expression level of any given transcript is determined by the number of sequencing reads that map on that specific transcript. However, the expression levels determined in a sequencing experiment can be influenced by several factors, such as, in particular, by the depth of the sequencing coverage obtained for the whole transcriptome. Thus, it is crucial to set a Detection Threshold (DTh), which represents the minimum number of reads mapping on a given transcript that is necessary to consider that gene as significantly expressed.

A Detection Threshold (DTh), based on the geometric distribution of the number of sequencing reads, was calculated as described in Materials and Methods (Statistical and Bioinformatic Data Analysis section). The DTh was determined independently on each of the samples sequenced: the DTh obtained for the total RNA sample was fixed at 20 reads (Additional file 3: Figure S3); thus, only those genes covered by at least 20 reads were considered expressed at significant levels in the conditions tested. In the total RNA samples, the number of CDS above DTh was 573, meaning that, from a sequencing run yielding 40 millions reads, only about 10% of the total BtE264 CDSs could be analysed; thus, in the absence of any rRNA removal treatment, it would be necessary to produce more than 400 millions of reads in order to reach a number of reads above Dth for all BtE264 CDS.

**Figure 2:**
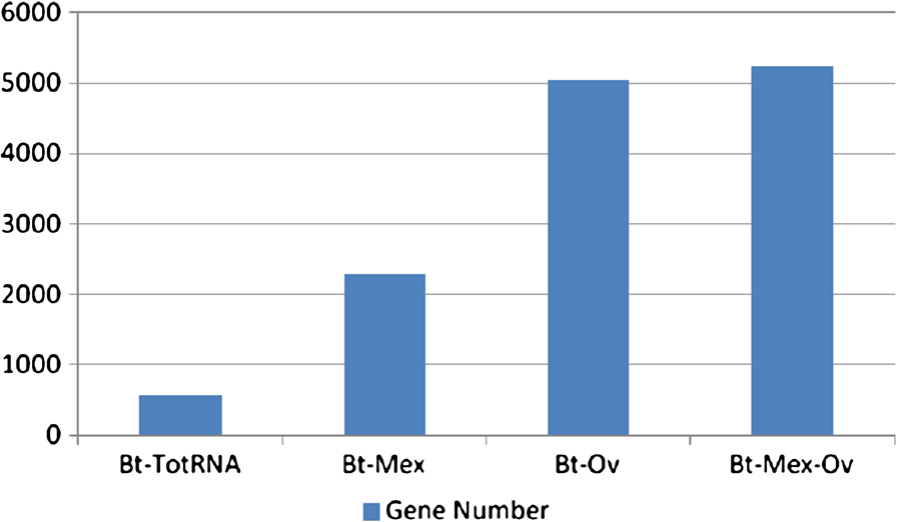
**Improvement in mRNA detection sensitivity.** Detection sensitivity increase at gene level of the two methods applied separately and in combination compared to the control sample (totRNA). The number of CDSs covered by more than 20 reads (Detection Threshold fixed for the total RNA) in control sample (Bt-totRNA) and in samples treated with the two rRNA removal methods separately and in combination (Bt-Mex; Bt-Ov; Bt-Mex-Ov) is represented by the histogram bars.

As shown in Figure 2, any of the mRNA enrichment treatments resulted in an increase of the number of CDS above DTh: in comparison to the 573 CDS (ca. 10% of total number of CDS in the BtE264 genome), CDSs above DTh increase to 2284 (about 40% of total CDS) in Mex-treated samples, while in samples treated either with Ovation or with the Mex-Ov more than 5000 genes, (about 90% of tot CDS), were found to be above DTh.

The increase in mRNA detection sensitivity was calculated by comparing the proportion of total mapped mRNA reads between the control and each rRNA depletion treatment (see Materials and Methods section, Statistical and Bioinformatic Data Analysis); for the Mex-Ov double treatment this resulted in more than 770% increase compared to the total RNA sample. Such increased sensitivity allows for a corresponding reduction in the number of sequencing reads necessary for the complete analysis of whole transcriptome expression profiling.

Indeed, it is possible to calculate the increase of efficiency in transcriptome coverage of each treatment alone or in combination by comparing the number of total reads necessary to target all the BtE264 CDSs. The Ov and Mex-Ov treatments allow the detection of almost all the CDSs with only about 10 million reads, with the Mex treatment only 50% of transcripts were covered with 28 millions reads, and in the control sample only 10% of CDSs were targeted with more than 40 millions reads. As a consequence, to cover all the CDSs in samples treated with only Mex more than 56 millions reads should be necessary and more than 400 millions reads should be produced to sequence the transcriptome in totRNA samples. The double treatments allows a scaling down of sequencing, in terms of total reads, by 40-fold, with respect to the totRNA sequencing, and by 5.6-fold with respect to the Mex treated samples sequencing, thus dramatically reducing the costs of sequencing.

### Robustness of mRNA relative abundance

**Figure 3:**
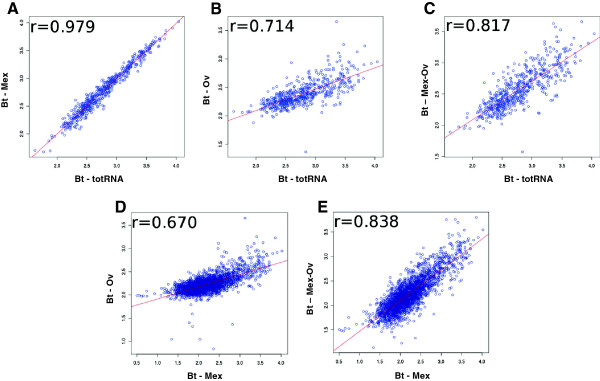
**Robustness of mRNA relative abundance.** Double-log scatter plots of RPKM values reporting the Pearson correlation coefficient showing the bias introduced by the two rRNA removal methods separately and in combination in mRNA relative abundance evaluation. On the X-axis of Figures 3A, 3B and 3C the log_10_ of RPKM values on the 573 transcripts above Dth in control samples (totRNAs)is reported, on the X-axis of Figure 3D and 3E the log_10_ of RPKM values for the 2368 .transcripts above Dth in Mex treated samples is reported; on the Y-axis of all the figures the log_10_ of RPKM values for the same transcripts subgroups in the samples treated with MICROBExpress (**A**) Ovation (**B**) combined treatment (**C**) respectively are reported.

An important criterion in validating mRNA enrichment methodologies is the evaluation of the potential bias introduced by the rRNA removal treatment on absolute and relative gene expression levels; this bias could derive for example from undesired removal of mRNA subsets. To assess the possible bias introduced in mRNA relative abundance, we evaluated the correlation of mRNA expression patterns between the control sample (*i.e.*, total RNA) and the samples subjected to Mex, Ov, and Mex-Ov treatments. Figure 3 shows Pearson’s correlation plots obtained, for the 573 CDS transcripts above the DTh, comparing the control samples (total RNA) to the treated samples, namely Mex (Figure 3A), Ov (Figure 3B) and Mex-Ov (Figure 3C). On the X-axis the log10 of the RPKM value for each transcripts above Dth in control samples (totRNAs) and on the Y-axis the log_10_ of RPKM value for the same transcripts in the samples treated is reported.

While samples only treated with the MICROBExpress kit showed very high correlation with total RNA (r = 0.979), treatment with the Ovation kit reduced the correlation with the control (r = 0.714), suggesting that the selective amplification methodology results in the introduction of a quite significant bias in mRNA relative abundance. However, the combination of the Ovation kit with MICROBExpress appears to overcome, albeit partially, the bias introduced by the Ovation alone, resulting in an improved correlation coefficient with the total RNA sample (r = 0.817) (Figure 3C).

As the correlation of mRNA expression patterns of the treated samples, in comparison to the control, had only taken into account 573 CDS, and considering that the Pearson’s correlation between the Mex treated samples and the control samples was very high (0.979), we decided to evaluate also the correlation of the Ov and Mex-Ov treatments against the mRNA expression patters obtained only after Mex treatment, thus correlating a wider number of transcripts (2368 CDS). As shown in Figure 3D and 3E this correlation decreases down to r=0.670 for the Ov and increases up to r=0.838 for the double treatment. Concerning the Ov treatment, we noticed a decrease of the variability range of mRNAs relative abundances as evidenced by the flattening of the regression line, probably due to the enrichment method based on retro-transcription using primers designed to avoid amplification of rRNAs sequences rather than completely random primers.

We can thus infer that the combination of the two treatments results in a significant improvement of mRNA detection sensitivity, allowing the expression analysis of almost all the BtE264 CDS with a low number of total reads, and at the same time does not introduce a consistent bias on their relative level of expression.

### Evaluation of mRNA differential expression profile

In order to estimate possible bias introduced by the rRNA removal treatments on mRNA differential expression profiles, the fold-change in gene expression levels of BtE264 samples grown in two different conditions was evaluated. We chose to analyze the effect of the anoxic stress on *B. thailandensis* by comparing bacterial cultures grown at 37°C with vigorous shaking (fully aerated conditions) versus no shaking (oxygen-limiting condition).

Total RNA was prepared in duplicate and each replicate from both conditions was subjected to MICROBExpress alone or to the combined method Mex-Ov because these are the treatments that introduced the lowest bias on mRNA relative abundance. The genes found to be above DTh in both samples (DTh = 18 reads; significantly expressed genes = 2368) were analysed with the DESeq pipeline to determine the statistically significant differentially expressed genes and derive their Fold Change (FC) values.

**Figure 4:**
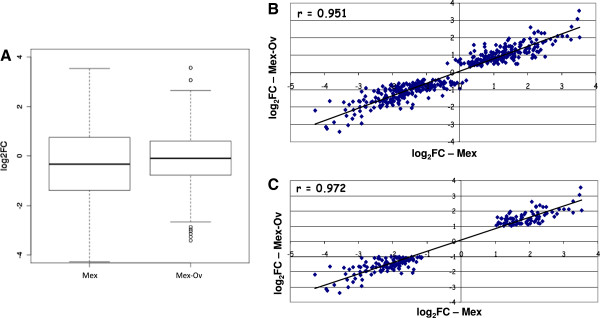
**Evaluation of mRNA differential expression profile.** The box plots distribution of the log_2_FC of DEGs from the comparison of RNAs, extracted from bacterial cultures grown at 37°C with vigorous shaking versus no shaking, and treated with MICROBExpress and with the combined method (**A**). Correlation of log_2_FC values of the 408 DEGs with p-val<0.01 (**B**), and of the 195 DEGs with p-val<0.01 and log_2_FC>1 or log_2_FC<−1 (**C**).

As shown in Figure 4A, the distribution of the log_2_FC of all the 2368 genes above DTh in the two growth conditions is less wide in samples treated with the Mex-Ov combined treatment than in samples treated only with MICROBExpress, evidencing reduced variability in fold change values, and reflecting part of the bias introduced by the Ov treatment. However, further correlation analysis of log_2_FC values of statistically significant differentially expressed genes (DEGs) showed very high correlation between the samples subjected to the combined rRNA removal treatment with respect to the simple MICROBExpress one. In particular, correlating the 408 DEGs with p-val<0.01, we obtained a Pearson’s correlation coefficient of 0.951 (Figure 4B), but, when this set was restricted to only those DEGs with a p-val<0.01 and either log_2_FC>1 or log_2_FC<−1 (*i.e.* 195 genes showing more than 2-fold change in expression levels), a Pearson’s correlation coefficient of 0.972 was obtained (Figure 4C). Thus, our results strongly suggest that Mex-Ov double treatment does not introduce any bias in log_2_FC of genes differentially expressed. Such a high Pearson correlation coefficient for differentially expressed genes is extremely important when a differential transcriptome analysis is performed, and counterbalances the bias introduced at the level of mRNA relative abundance (see Figure 3C).

## Discussion

In this work, we have developed a method for rRNA removal deriving from the combination of two commercially available kits: the MICROBexpress kit from Ambion and the Ovation kit from NuGEN, based respectively on rRNA capture by specific oligonucleotides and on selective amplification using random primers that do not recognize bacterial rRNA sequences. To validate this method, we chose the bacterium *B. thailandensis*, because of the complexity (6.72 Mbp) and of the high GC content (67.7%) of its genome. Indeed, genomes with high GC contents constitute a problem in transcriptomic studies, since they reduce the efficiency of most steps in the preparation of sequencing libraries.

We found that the Ovation kit was much more effective then MICROBexpress in rRNA removal; however, combined MICROBexpress/Ovation treatment was crucial to reduce the bias in mRNA relative abundance, thus preserving differential expression profile. The combination of the two methods results in an mRNA enrichment of more than 238-fold enabling the coverage of almost all (more than 90%) *B. thailandensis* transcripts with less than 10 million sequencing reads. Therefore using this combined removal method it could be possible to multiplex up to 25 different bacterial transcriptomes, of the same size and complexity as BtE264 transcriptome, in a single GAIIx run (1 Illumina flow cell), thus resulting in a significant reduction in sequencing costs.

Several recently published papers describe various rRNA removal methods for Prokaryotic RNA sequencing improving the performance in rRNA removal, in comparison to standard kits, that have been the most widely used procedure for rRNA removal so far [15,20]. The method proposed in our work shows comparable or even better efficiency in mRNA enrichment with respect to the previous works. In terms of mRNA fidelity conservation, our approach appears to be more effective on GC-rich genomes than other methods such as treatment with Duplex-specific nuclease, which can result in significant bias in mRNA abundance quantification [19].

A recent work by Giannoukos et al. [13], comparing 5 different rRNA removal methods, is the most exhaustive comparative analysis performed both on single microorganisms and on faecal samples to date. These authors report very good results, both in terms of rRNA depletion and of robustness of mRNA profiles, using the Ribo-Zero rRNA removal kit. Our results obtained by using the MICROBexpress kit alone are comparable with those of Giannoukos et al. [13] both in terms of rRNA depletion (more than 96% rRNA residual in our study, more than 90% in Giannoukos paper) and robustness of mRNA relative abundance respect to totRNA (R^2^= 0.979 in our study and R^2^= 0.90 in Giannoukos paper, respectively).

It should be noted that good rRNA removal in GC-rich bacteria with the Ovation kit obtained in our experimental conditions (rRNA amount decreased to an average of 61%), is in contrast with the results of Giannoukos et al. (residual rRNA about 90%), although the correlation between Ov-treated samples and control RNA is similar in both studies. However, in our study, we prepared the sequencing libraries using the ENCORE kit from NuGEN, since it is the most compatible with the size of cDNAs synthesized by the random primer mix selectively designed to enrich the mRNA portion of bacterial total RNA.

The Ovation prokaryotic RNA–seq system kit is not compatible with the standard Illumina RNA library preparation kit, because this protocol makes a size selection of fragments above 200bp length, but it can be made compatible with a modified version of this Illumina protocol by changing the size selection step. However, the same protocol modification should have been applied also to the samples treated solely with MICROBexpress. The selection of fragments of 200bp size, in the Illumina library preparation protocol, might lead to a loss of the mRNA enriched during the retro-transcription with the not-so random primer mix of the Ovation kit, which generates cDNAs fragments of about 150bp. Thus, the residual rRNA portion, which is made of repeated sequences, could be preferentially amplified during the PCR step of library preparation. Probably the low performance of the Ovation kit observed by Giannoukos does not derive from the rRNA removal step, but it could be a bias introduced by the Illumina library preparation method and, in particular, by the size selection step followed by preferential amplification of residual ribosomal RNA. Therefore, we preferred to use the most compatible kit with the Ovation treatment, *i.e.*, the ENCORE library preparation from NuGEN, without modifying the Illumina protocol.

To reduce the bias introduced by this selective priming step we coupled the Ov treatment with a previous removal step by Mex treatment.

We compared results obtained from rRNA removal protocols based on different chemistries (capture with probes against retro-transcription with selective random primers) and from libraries prepared with different protocols (Illumina TruSeq RNA libraries against NuGEN’s Encore™ NGS libraries) in order to really evaluate the efficiency of the combined method here proposed and to actually test the robustness of the whole protocol respect to the MICROBExpress that, till now, has been the most widely used procedure for rRNA removal.

This combined rRNA removal method was applied to enrich mRNAs in two highly GC rich microorganisms: *Streptosporangium roseum* and *Amycolatopsis mediterranei*, both having large genomes with more than 70% GC content. We decreased the rRNA content, after the combined treatment, to 66% and to 70%, thus obtaining an mRNA enrichment of 68-fold and 60-fold respectively (unpublished data), and mRNA relative abundance correlation to totRNA similar to that obtained with *B. thailandensis*.

## Conclusions

We believe that the MICROBExpress/Ovation combined method described here could be worthy of consideration for RNAseq projects that must be carried out in GC-rich bacteria, and that it could be a possible alternative to other valid methods. Indeed, the combination of MICROBExpress and Ovation highly increases the mRNA detection sensitivity and does not introduce any significant bias on relative mRNA abundance, allowing a reliable analysis of differentially expressed genes.

In contrast, our results suggest that the Ovation kit alone might introduce significant bias in mRNA relative abundance. Thus, we can conclude that the combined method can be valid for RNA sequencing of whole transcriptomes of microorganisms with high GC content and complex genomes enabling at the same time an important scaling down of sequencing costs.

## Methods

### Bacterial growth and RNA extraction

The bacterium utilized in this study is *Burkholderia thailandensis* strain E264 [21]. *B. thailandensis* E264 was grown in LB medium [22] at 37°C for 16 hours either in full aeration, achieved by constant shaking at 100 rpm/minute, or in oxygen-limiting conditions (no shaking). Total RNA was extracted from 1-ml culture samples using the GeneElute™ total RNA Purification Kit (SIGMA); RNA was recovered in 50 μl of Elution Solution.

Total RNA samples were treated with Recombinant DNase I (Ambion) to remove genomic DNA contamination. Two units of DNase I were added for up to 10 μg RNA in a 50 μl reaction. The reaction was incubated at 37°C for 10 minutes; afterwards RNA was purified by precipitation at −80°C for 2 hours by adding 0.1 volume 3M sodium acetate, 5 μg glycogen and 3 volume 100% ethanol.

After extraction and DNase treatment, total RNA samples were quantified with a NanoDrop spectrophotometer (NanoDrop Technologies) and analyzed by capillary electrophoresis on an Agilent Bioanalyzer (Agilent), using the RNA 6000 Nano LabChip Kit. The RNA samples showing an RIN (RNA Integrity Number, a quality parameter calculated by the instrument software) value higher than 8 were processed.

### rRNA removal treatments, library construction and sequencing

rRNA removal from *B. thailandensis* E264 total RNA through subtractive hybridization using the MICROBExpress. Bacterial mRNA Enrichment Kit (Ambion, Austin, Texas) was performed according to the manufacturer’s protocol. Briefly, total RNA (5 μg) was purified and incubated with the capture oligonucleotide mix in binding buffer for 30 minutes. Magnetic beads loaded with an oligonucleotide sequence able to hybridize to the unpaired region of the capture oligonucleotide were added to the mixture and allowed to hybridize for 15 minutes in order to form a ternary complex with the capture oligonucleotide and the rRNAs molecules. The beads were pulled to the side of the tube with a magnet and the unbound RNA in the solution was moved to a fresh tube. The magnetic beads were briefly washed to ensure full recovery of the unbound RNA; the unbound RNA was pooled and precipitated with ethanol. rRNA removal after each treatment was visually assessed by Agilent 2100 Bioanalyzer with the RNA 6000 Nano LabChip Kit. mRNA enriched from MICROBExpress Bacterial mRNA Enrichment Kit and total RNA (500 ng) were used for the preparation of Illumina TruSeq RNA libraries, following manufacturer’s instructions.

For removal of rRNA by selective amplification, total RNA (500 ng) was retro-transcribed using the Ovation Prokaryotic RNA-seq System, which uses random primers selectively designed to avoid rRNA amplification. After first strand synthesis double stranded cDNA is obtained and about 200 ng were used to prepare NGS library with NuGEN’s Encore™ NGS Library System, according to the manufacturer’s protocol. Due to small size of cDNA obtained no fragmentation treatment was performed prior to library preparation, as suggested by NuGEN’s instructions. For double treatment (subtractive hybridization and selective amplification of mRNA), 500 ng of mRNA-enriched from MICROBExpress kit were used for the cDNA synthesis by Ovation Prokaryotic RNA-seq System. Each sample was prepared as a replicate and sequenced.

Sequencing was performed using the Illumina Genome Analyzer IIx platform to generate paired-end 48bp reads in the case of NuGEN’s Encore™ NGS Libraries and paired-end 86bp reads in the case of Illumina TruSeq RNA library.

### Nucleotide sequence accession numbers

Raw sequences reported in this article have been deposited in the NCBI Short Read Archive (SRA). Accession number: SRA062706.

### Statistical and bioinformatic data analysis

Raw reads generated by the sequencing run were extracted and divided into separated datasets according to Illumina index (de-multiplexed) using the GERALD script developed by Illumina.

For the samples prepared with NuGEN Encore™ NGS kit, two mate reads (read 1 and read 2) were linked and de-multiplexed with fastx-toolkit package (fastx_barcode_splitter.pl) [23]. In order to filter out the reads whose sequence contained part of the Illumina adaptor (Illumina adaptor contaminated reads) fastx-mcf software [24] was used.

All the samples were mapped against the *Burkholderia thailandensis* strain E264 genome (Ref Seq accession number: NC_007651.1 and NC_007650.1; Gen Bank accession number: CP000086.1 and CP000085.1) using BWA software [25] allowing up to four mismatches. The unmapped reads coming from the NuGEN Encore™ NGS libraries, were mapped with BLAST [26], against the *B. thailandensis* E264 genome, then all the reads with less than 90% of identity and less than 25 nucleotide matching were filtered out. The reads mapped with BWA and those mapped with BLAST were merged in a unique BAM alignment file.

The statistical analysis for rRNA depletion and mRNA enrichment were performed with BEDTools [27] using as input the GFF file available from GenBank, containing the annotation of *B. thailandensis* E264 genome.

In RNA-seq experiments, to define background noise and thus discriminate between random and real expression values, it is fundamental to find out those transcripts that are covered by a number of reads too low to be considered proportional to their level of expression.

The definition of the background can be achieved by determining the Detection Threshold (DTh). The DTh was calculated by plotting the read depth (x-axis) and the log10 number of transcript covered by a given read depth (y-axis).

It can be assumed that reads sampled randomly from the genome follow a geometric distribution, but in a RNA-seq experiment the distribution of the reads per transcript should be proportional to that transcript level of expression.

Assuming that the number of reads per transcript follows a null distribution represented by:

$$P\left(X=k\right)=p^{k-1}\left(1-p\right)$$

from this distribution we can derive that *P*(*X=k*) is linear in k. Using this hypothesis we can estimate the read depth threshold by plotting the linear fitting line for the reads per transcript distribution for each control sample (totRNA). Since our samples are not randomly distributed, the transcripts covered by a number of reads below the Detection Threshold were excluded from the analysis.

The improvement in mRNA detection sensitivity was calculated as described previously by He et al. [28] by comparing the proportion of total mapped mRNA reads between the control and each treatment. The Pearson correlations of mRNA expression patterns between the samples were calculated using the log_10_ of RPKM [29]. The R package DESeq was used to analyse count data from RNA-Seq sequencing assays and to identify differentially expressed genes (DEGs) [30]; statistical analysis and correlation plots have been produced using R version 2.13.2 software (http://www.R-project.org/). DESeq was used because it is the simplest available tool for gene expression analysis; it doesn’t need a gene annotation table (.gtf file) for transcripts recostruction, and we believe that this feature can make DESeq particularly suitable for bacterial RNA-Seq data analysis respect to other tools such as for example Cufflinks.

## Competing interests

The authors declare that they have no competing interests.

## Authors’ contributions

CP conceived the design of the study, carried out the samples processing and sequencing experiments, wrote the manuscript; AP participated in the design of the study, performed the bioinformatic and statistical Data Analysis, helped to draft the manuscript; CC participated in the design of the study, carried out the samples processing and sequencing experiments, helped to draft the manuscript; ER carried out the cell growths, participated to the Data Analysis, helped to draft the manuscript; LP participated in the design of the study, participated to the Data Analysis; LT carried out the cell growths, performed the RNA extractions; GDB participated in the design of the study, coordinated the efforts; PL participated in the design of the study, wrote the manuscript, coordinated the efforts. All authors read and approved the final manuscript.

## Supplementary Material

Additional file 1 **Figure S1.** The Agilent Bioanalyzer profiles of:
▪ totRNAs and Mex-RNAs
▪ the Illumina sequencing libraries of Mex samples (DNA High Sensitivity assay)
▪ the sequencing libraries of the samples treated with the Mex-Ov combined treatment and with the Ov treatment alone, prepared with the NuGEN’s Encore™ NGS system (DNA High Sensitivity assxay). Click here for file

Additional file 2 **Figure S2.** Pearson’s correlation plots showing the reproducibility of Illumina sequencing on samples treated with the rRNA removal combined method; each sample was prepared as a replicate and sequenced in two separate Illumina Runs. Click here for file

Additional file 3 **Figure S3.** Graphical representation of the reads count frequencies in control sample (tot-RNA) for the Detection Threshold determination; on the X-axis the number of read mapping on each CDS, on the Y-axis the log10 of the read count. Click here for file

## References

[B1] Arraiano CM Andrade JM Domingues S Guinote IB Malecki M Matos RG Moreira RN Pobre V Reis FP Saramago M The critical role of RNA processing and degradation in the control of gene expression FEMS Microbiol Rev 2010 34 883 923 20659169 10.1111/j.1574-6976.2010.00242.x

[B2] Passalacqua KD Varadarajan A Ondov BD Okou DT Zwick ME Bergman NH Structure and complexity of a bacterial transcriptome J Bacteriol 2009 191 3203 3211 10.1128/JB.00122-09 19304856 PMC2687165

[B3] Perkins TT Kingsley RA Fookes MC Gardner PP James KD Yu L Assefa SA He M Croucher NJ Pickard DJ A strand-specific RNA-Seq analysis of the transcriptome of the typhoid bacillus *Salmonella typhi* PLoS Genet 2009 5 e1000569 10.1371/journal.pgen.1000569 19609351 PMC2704369

[B4] Yoder-Himes DR Chain PS Zhu Y Wurtzel O Rubin EM Tiedje JM Sorek R Mapping the *Burkholderia cenocepacia* niche response via high-throughput sequencing Proc Natl Acad Sci USA 2009 106 3976 3981 10.1073/pnas.0813403106 19234113 PMC2645912

[B5] Passalacqua KD Varadarajan A Byrd B Bergman NH Comparative transcriptional profiling of *Bacillus cereus sensu lato* strains during growth in CO2-bicarbonate and aerobic atmospheres PLoS One 2009 4 e4904 10.1371/journal.pone.0004904 19295911 PMC2654142

[B6] Croucher NJ Fookes MC Perkins TT Turner DJ Marguerat SB Keane T Quail MA He M Assefa S Bahler J A simple method for directional transcriptome sequencing using Illumina technology Nucleic Acids Res 2009 37 e148 10.1093/nar/gkp811 19815668 PMC2794173

[B7] Filiatrault MJ Stodghill PV Bronstein PA Moll S Lindeberg M Grills G Schweitzer P Wang W Schroth GP Luo S Transcriptome analysis of *Pseudomonas syringae* identifies new genes, noncoding RNAs, and antisense activity J Bacteriol 2010 192 2359 2372 10.1128/JB.01445-09 20190049 PMC2863471

[B8] Beaume M Hernandez D Farinelli L Deluen C Linder P Gaspin C Romby P Schrenzel J Francois P Cartography of methicillin-resistant S. aureus transcripts: detection, orientation and temporal expression during growth phase and stress conditions PLoS One 2010 5 e10725 10.1371/journal.pone.0010725 20505759 PMC2873960

[B9] Gilbert JA Field D Huang Y Edwards R Li W Gilna P Joint I Detection of large numbers of novel sequences in the metatranscriptomes of complex marine microbial communities PLoS One 2008 3 e3042 10.1371/journal.pone.0003042 18725995 PMC2518522

[B10] Poretsky RS Hewson I Sun S Allen AE Zehr JP Moran MA Comparative day/night metatranscriptomic analysis of microbial communities in the North Pacific subtropical gyre Environ Microbiol 2009 11 1358 1375 10.1111/j.1462-2920.2008.01863.x 19207571

[B11] Dodd D Moon YH Swaminathan K Mackie RI Cann IK Transcriptomic analyses of xylan degradation by *Prevotella bryantii* and insights into energy acquisition by xylanolytic bacteroidetes J Biol Chem 2010 285 30261 30273 10.1074/jbc.M110.141788 20622018 PMC2943253

[B12] Stewart FJ Ottesen EA DeLong EF Development and quantitative analyses of a universal rRNA-subtraction protocol for microbial metatranscriptomics ISME J 2010 4 896 907 10.1038/ismej.2010.18 20220791

[B13] Giannoukos G Ciulla DM Huang K Haas BJ Izard J Levin JZ Livny J Earl AM Gevers D Ward DV Efficient and robust RNA-seq process for cultured bacteria and complex community transcriptomes Genome Biol 2012 13 r23 10.1186/gb-2012-13-3-r23 22455878 PMC3439974

[B14] Sharma CM Hoffmann S Darfeuille F Reignier J Findeiss S Sittka A Chabas S Reiche K Hackermuller J Reinhardt R The primary transcriptome of the major human pathogen Helicobacter pylori Nature 2010 464 250 255 10.1038/nature08756 20164839

[B15] Wurtzel O Sapra R Chen F Zhu Y Simmons BA Sorek R A single-base resolution map of an archaeal transcriptome Genome Res 2010 20 133 141 10.1101/gr.100396.109 19884261 PMC2798825

[B16] Vivancos AP Guell M Dohm JC Serrano L Himmelbauer H Strand-specific deep sequencing of the transcriptome Genome Res 2010 20 989 999 10.1101/gr.094318.109 20519413 PMC2892100

[B17] Jager D Sharma CM Thomsen J Ehlers C Vogel J Schmitz RA Deep sequencing analysis of the *Methanosarcina mazei* Go1 transcriptome in response to nitrogen availability Proc Natl Acad Sci USA 2009 106 21878 21882 10.1073/pnas.0909051106 19996181 PMC2799843

[B18] Irnov I Sharma CM Vogel J Winkler WC Identification of regulatory RNAs in *Bacillus subtilis* Nucleic Acids Res 2010 38 6637 6651 10.1093/nar/gkq454 20525796 PMC2965217

[B19] Yi H Cho YJ Won S Lee JE Jin Yu H Kim S Schroth GP Luo S Chun J Duplex-specific nuclease efficiently removes rRNA for prokaryotic RNA-seq Nucleic Acids Res 2011 39 e140 10.1093/nar/gkr617 21880599 PMC3203590

[B20] Galyov EE Brett PJ DeShazer D Molecular insights into *Burkholderia pseudomallei* and *Burkholderia mallei* pathogenesis Annu Rev Microbiol 2010 64 495 517 10.1146/annurev.micro.112408.134030 20528691

[B21] Kim HS Schell MA Yu Y Ulrich RL Sarria SH Nierman WC DeShazer D Bacterial genome adaptation to niches: divergence of the potential virulence genes in three Burkholderia species of different survival strategies BMC Genomics 2005 6 174 10.1186/1471-2164-6-174 16336651 PMC1343551

[B22] Sambrook J Russell DW Molecular Cloning. A Laboratory Manual 2001 Cold Spring Harbor, New York: Cold Spring Harbor Laboratory Press

[B23] Pearson WR Wood T Zhang Z Miller W Comparison of DNA sequences with protein sequences Genomics 1997 46 24 36 10.1006/geno.1997.4995 9403055

[B24] ea-utils: “Command-line tools for processing biological sequencing data” [http://code.google.com/p/ea-utils]

[B25] Li H Durbin R Fast and accurate short read alignment with Burrows-Wheeler transform Bioinformatics 2009 25 1754 1760 10.1093/bioinformatics/btp324 19451168 PMC2705234

[B26] Altschul SF Gish W Miller W Myers EW Lipman DJ Basic local alignment search tool J Mol Biol 1990 215 403 410 2231712 10.1016/S0022-2836(05)80360-2

[B27] Quinlan AR Hall IM BEDTools: a flexible suite of utilities for comparing genomic features Bioinformatics 2010 26 841 842 10.1093/bioinformatics/btq033 20110278 PMC2832824

[B28] He S Wurtzel O Singh K Froula JL Yilmaz S Tringe SG Wang Z Chen F Lindquist EA Sorek R Hugenholtz P Validation of two ribosomal RNA removal methods for microbial metatranscriptomics Nat Methods 2010 7 807 812 10.1038/nmeth.1507 20852648

[B29] Mortazavi A Williams BA McCue K Schaeffer L Wold B Mapping and quantifying mammalian transcriptomes by RNA-Seq Nat Methods 2008 5 621 628 10.1038/nmeth.1226 18516045 PMC13303166

[B30] Anders S Huber W Differential expression analysis for sequence count data Genome Biol 2010 11 R106 10.1186/gb-2010-11-10-r106 20979621 PMC3218662

