# Supplementary figures and images for: An efficient rRNA removal method for RNA sequencing in GC-rich bacteria
Source: Microb Inform Exp. 2013 Jan 7;3:1. doi: 10.1186/2042-5783-3-1 (PMC3563489; doi:10.1186/2042-5783-3-1)

Fig. S1

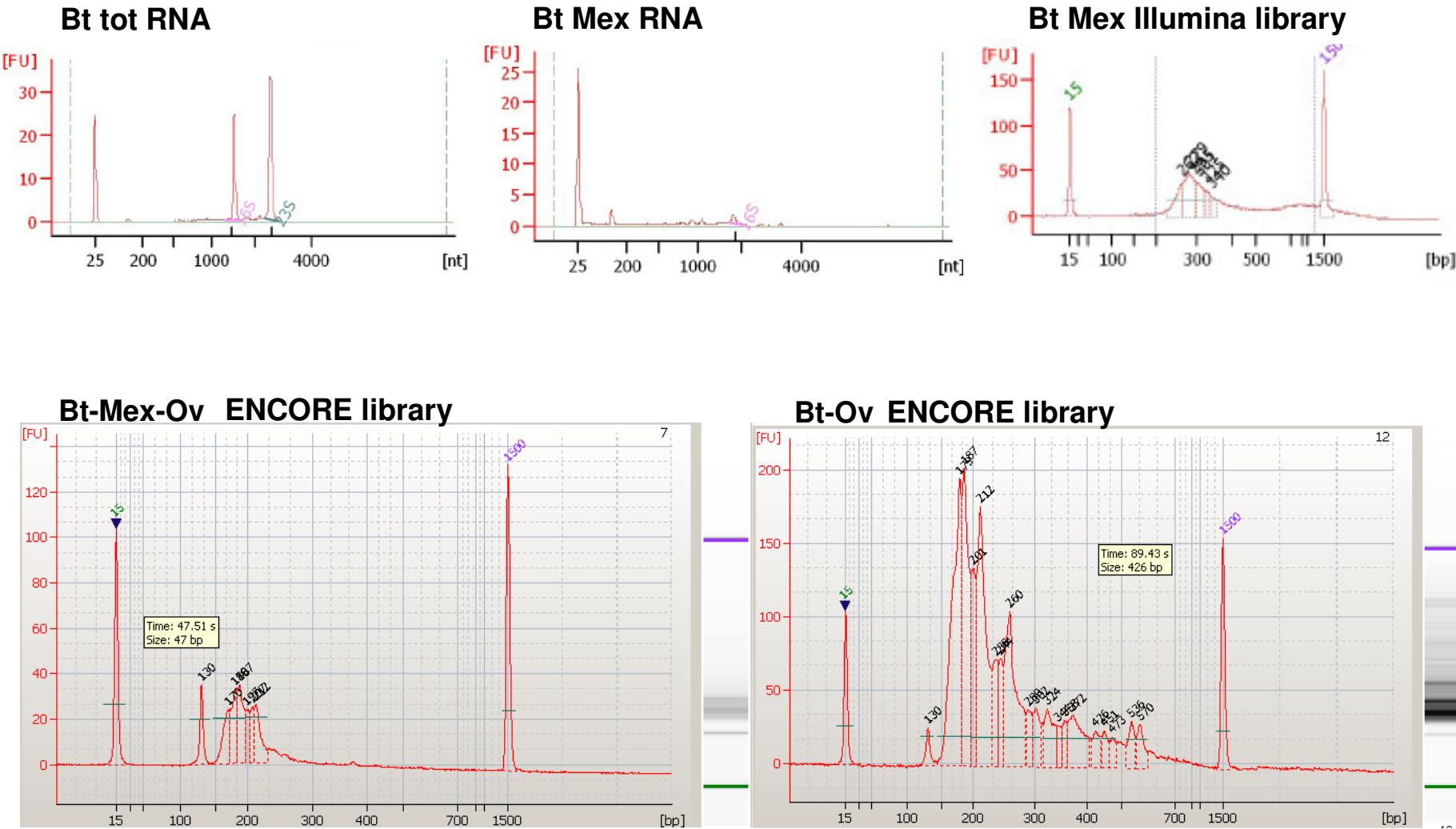

Supplement: Additional file 1 — Figure S1. The Agilent Bioanalyzer profiles of: ▪ totRNAs and Mex-RNAs ▪ the Illumina sequencing libraries of Mex samples (DNA High Sensitivity assay) ▪ the sequencing libraries of the samples treated with the Mex-Ov combined treatment and with the Ov treatment alone, prepared with the NuGEN’s Encore™ NGS system (DNA High Sensitivity assxay). [file 2042-5783-3-1-S1.pdf]

Fig. S2

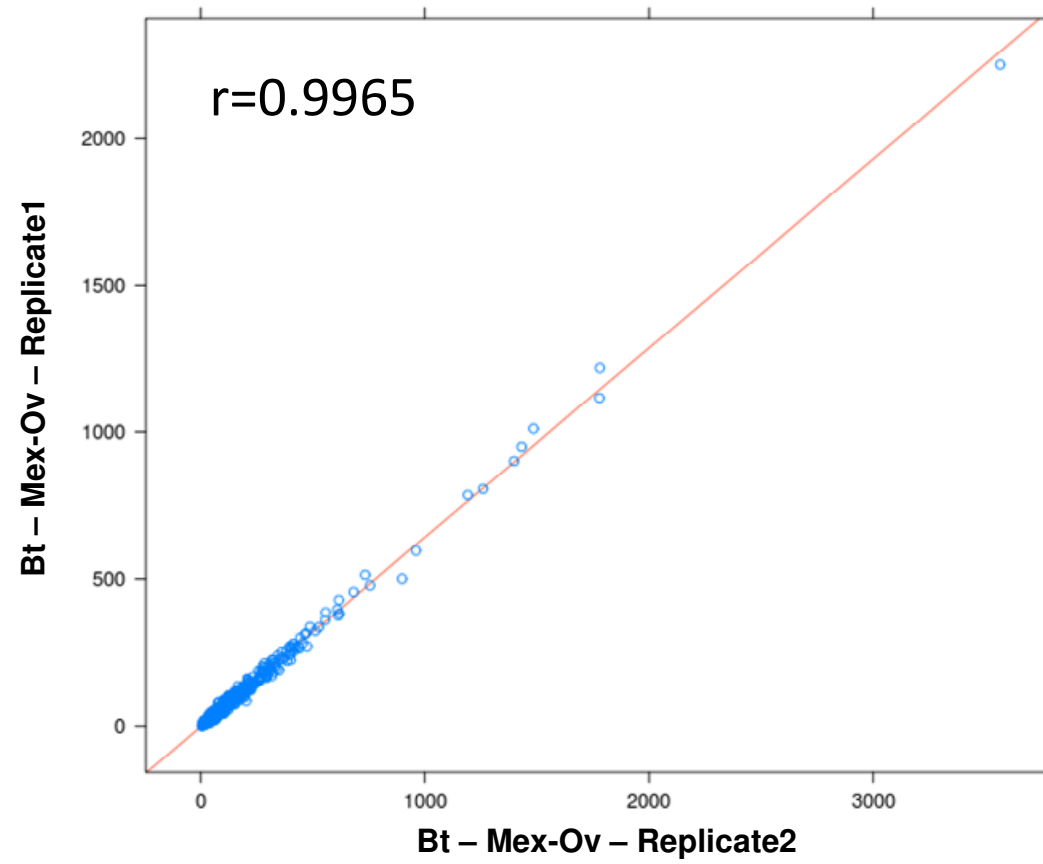

Supplement: Additional file 2 — Figure S2. Pearson’s correlation plots showing the reproducibility of Illumina sequencing on samples treated with the rRNA removal combined method; each sample was prepared as a replicate and sequenced in two separate Illumina Runs. [file 2042-5783-3-1-S2.pdf]

Fig. S3

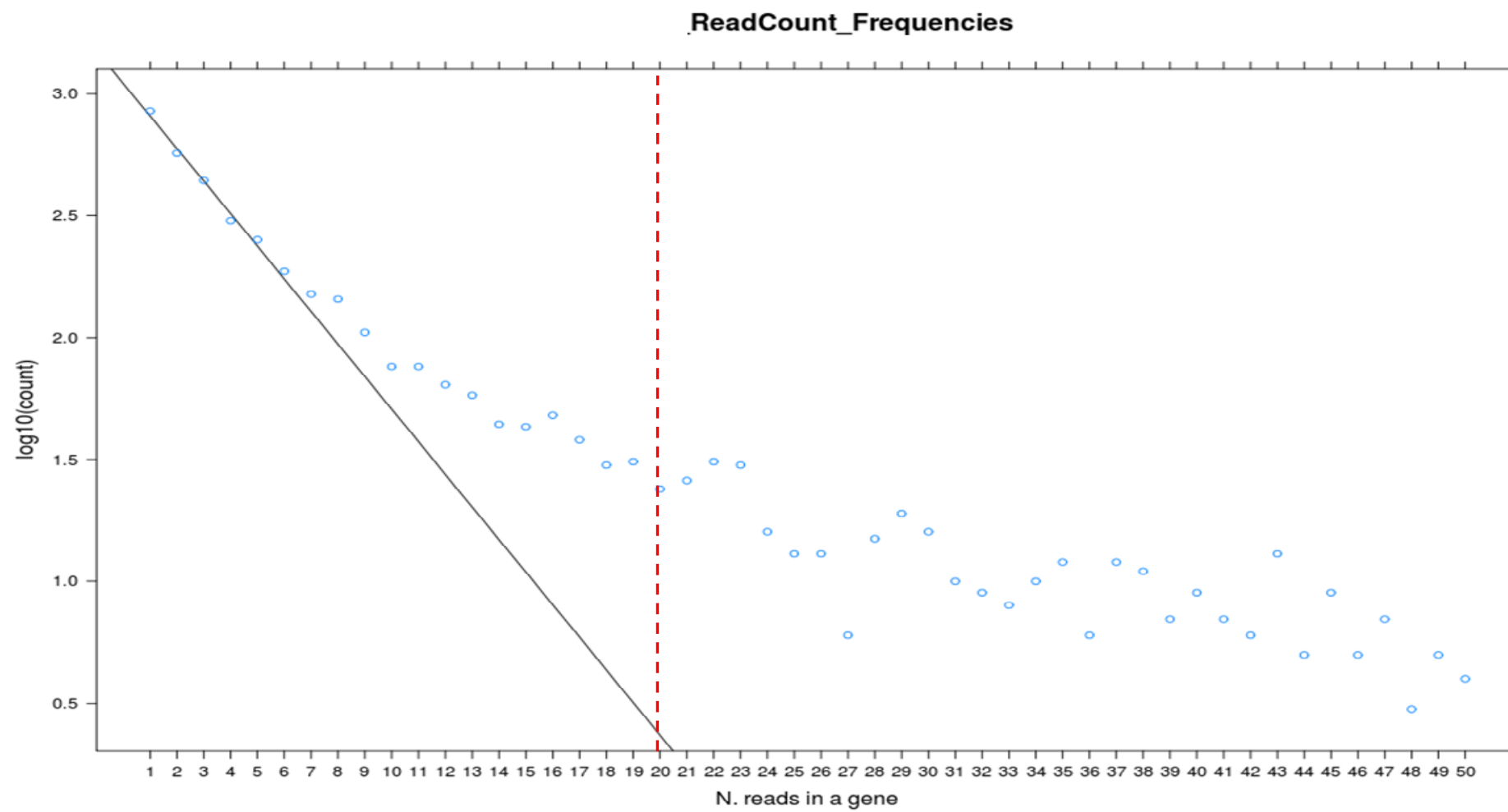

Supplement: Additional file 3 — Figure S3. Graphical representation of the reads count frequencies in control sample (tot-RNA) for the Detection Threshold determination; on the X-axis the number of read mapping on each CDS, on the Y-axis the log10 of the read count. [file 2042-5783-3-1-S3.pdf]
